# Trip score – A three-pillar risk stratification model proposal for antibiotic and immunomodulator coadministration

**DOI:** 10.3389/fphar.2026.1878471

**Published:** 2026-08-11

**Authors:** Hanna Sebesi, Erika Bán, László-István Bába, Adrian Man

**Affiliations:** 1 Department of Pharmacology and Clinical Pharmacology ME1, George Emil Palade University of Medicine, Pharmacy, Sciences and Technology of Târgu Mureş, Târgu Mures, Romania; 2 Preclinical Studies Laboratory, UMFST, Targu Mures, Romania; 3 The Organizing Institution for Doctoral University Studies (IOSUD) - Doctoral School of Medicine and Pharmacy, George Emil Palade University of Medicine, Pharmacy, Science, and Technology, Târgu Mureș, Romania; 4 Department of Pharmacology and Clinical Pharmacy, George Emil Palade University of Medicine, Pharmacy, Sciences and Technology of Târgu Mureş, Târgu Mures, Romania; 5 Department of Microbiology, George Emil Palade University of Medicine, Pharmacy, Sciences and Technology of Târgu Mureş, Târgu Mures, Romania

**Keywords:** antibiotics, clinical pharmacology, drug-drug interaction (DDI), immunomodulators, pharmacodynamic (PD), pharmacokinetic, pharmacomicrobiome, score system

## Abstract

**Background:**

Conventional drug-drug interaction checkers predominantly rely on a two-dimensional approach, including pharmacokinetic and pharmacodynamic mechanisms. Emerging recognition of the gut microbiome is often an overlooked parameter in drug interferences. This study introduces a novel three-pillar risk stratification scoring model, incorporating pharmacomicrobiomics, to help refine the interaction landscape between antibiotics and immunomodulators for safer and more precise patient care.

**Methods:**

Sixty-four antibiotic-immunomodulator drug pairs were analyzed across eight major drug classes. A standardized Risk Score was developed, assigning weighted values to Pharmacokinetic, PD and PM mechanisms based on pharmacological plausibility and clinical severity. This model’s findings were compared against established clinical classifications (Lexicomp®) to identify blind spots in traditional risk assessment.

**Results:**

The three-pillar model revealed 106 mechanistic interactions, representing a 65.5% increase in identified pathways compared to traditional two-dimensional models. The average mechanism per drug pair increased to 1.65. While standard databases categorized most pairings as moderate risk (Category C), our scoring system classified 51.5% (n = 33) as High-Risk (TRS ≥4). The PM pillar was pivotal in explaining metabolic interferences, such as antibiotic-induced dysbiosis, leading to either toxic surges in Tacrolimus levels or therapeutic failure of Mycophenolate mofetil due to disrupted enterohepatic recycling.

**Conclusion:**

Integrating pharmacomicrobiomics as the third pillar of drug interactions uncovers an invisible layer of clinical risk. Traditional tools significantly underestimate the burden of interactions by omitting simultaneous interferences and the role of the gut microbiome as a pre-systemic filter. While the scoring system presented here represents a novel proposal that requires future clinical validation, it provides a necessary framework for precise prescribing and enhancing patient safety in vulnerable multimorbid patients.

## 1 Introduction

The management of autoimmune diseases is defined by a central therapeutic paradox: the potent immunomodulatory agents required to achieve remission inevitably predispose patients to a heightened risk of infection. The magnitude of this susceptibility is well-documented; for instance, bacterial infections account for 55% of mortality in patients with Systemic Lupus Erythematosus (SLE) (Qiu et al., 2019). Similarly, among patients with psoriasis, systemic immunomodulatory therapy has been shown to nearly double the risk of bacterial infections (Hazard Ratio = 1.99) (Jian et al., 2025).

Antibiotics remain fundamental pillar of modern medicine, essential for the management of life-threatening infectious diseases, where the choice of agent dictates the clinical outcome. Concurrently, immunomodulatory agents are indispensable for survival and quality of life in autoimmune disorders.

To treat intercurrent bacterial infections, acute antimicrobial therapy is prescribed, which combined with chronic immunomodulatory agents creates not only a complex pharmacological environment, but also an additional risk factor for an already vulnerable patient group (Bhagat et al., 2021). This pharmacological intersection frequently leads to clinically significant drug-drug interactions (DDIs), where the acute antibiotic therapy alters the safety profile or efficacy of the immunosuppressant. Pharmacokinetic (PK) interactions primarily affect drug metabolism either through the inhibition or induction of the cytochrome P450 enzyme family, leading to supra- or subtherapeutic serum concentrations. This proves especially dangerous due to the narrow therapeutic index (NTI) of most immunosuppressants, where the window between therapeutic efficacy and toxicity is separated only by a thin margin (Narrow therapeutic index drugs, 2026). On the other hand, pharmacodynamic (PD) interactions result in additive toxicities, such as compounded myelosuppression or severe nephrotoxicity (Chellal et al., 2025; Choi et al., 2021; Li et al., 2024).

Beyond these interaction pathways, recent evidence highlights a third and emerging viewpoint on drug metabolism: pharmacomicrobiomics (PM), defined as the study of how the gut microbiome interacts with drugs, influencing efficacy, metabolism and toxicity, and how drugs in turn alter the commensal gut flora (Gronich et al., 2025). Sulfasalazine, for instance, is the prodrug of 5-aminosalicylic acid and sulphapyridine, that requires intestinal azoreductase-producing bacteria for activation. The efficiency of this cleavage is proportionate to the concentration of commensal flora, which can be significantly depleted during a course of antibiotics, diminishing the effect of sulfasalazine (Zhao et al., 2025). Additionally, enterohepatic recirculation is a major component of a drug’s half-life and bioavailability, that frequently requires the gut microbiome for the deconjugation of drugs or metabolites. Another example is mycophenolate mofetil since its plasma concentrations are highly dependent on this recycling process, hence antibiotic-induced dysbiosis inevitably leads to altered therapeutic levels (Guo et al., 2019).

The aim of this study is to bridge the existing gap between theoretical and clinical pharmacology by developing a comprehensive score-based risk assessment system to guide clinical decision making when fighting bacterial infections in autoimmune patients. The risk stratification matrix covers the three primary mechanistic pathways in drug metabolism: pharmacokinetics, pharmacodynamics and pharmacomicrobiomics.

## 2 Materials and methods

### 2.1 Search strategy and data collection

The identification of drug-drug interactions was conducted using the Lexicomp® Drug Interaction database (accessed via UpToDate®) as the primary search engine. Each of the eight selected immunomodulators served as index drugs to screen for every documented intersection with antibiotic agents.

The scoring system used by Lexicomp classifies interactions according to the following scale:

A = No Known Interaction; B = No Action Needed; C = Monitor Therapy; D = Consider Therapy Modification; X = Avoid Combination (Dom et al., 2025).

To mitigate selection bias and ensure a comprehensive analysis, identified interactions were cross-referenced with primary literature using PubMed. This verification allowed for an accurate classification of each interaction within the PK, PD or PM pillars. Finally, antibiotics were subsequently organized by their pharmacological class to assist a structured assessment of risk across the therapeutic groups.

### 2.2 Selection of therapeutic agents

Eight systemic immunosuppressant agents were selected for analysis based on their high prevalence in multiple autoimmune diseases: calcineurin inhibitors (cyclosporine, tacrolimus), antimetabolites (methotrexate, mycophenolate mofetil, azathioprine), cyclophosphamide, hydroxychloroquine and dapsone. These eight agents represent five different biochemical classes, making this tool more universal and inclusive.

This scoring system was developed exclusively for small molecular immunosuppressants (SMIs), biological therapeutic agents being excluded due to their distinct structural properties and pharmacokinetic interaction profile. SMIs are broken down by proteolytic degradation and are not metabolized via the standard cytochrome P450 or P-gp pathways (Ogihara et al., 2023).

The DDI search was performed using an Index-Drug Lead Strategy, identifying every antibiotic that creates variable intersections with our immunomodulatory agents.

### 2.3 Development of the scoring system

The risk stratification matrix is based on the three previously detailed pillars of drug metabolism: pharmacokinetic, pharmacodynamic, and pharmacomicrobiomic interactions. To ensure clinical reproductibility, a standardized numerical weight of +1, +2 and +3 was assigned based on the severity of the anticipated clinical outcome and magnitude of the interaction (summarized in Table 1). Specifically, scores were calibrated as follows: a +1 weight represents minor interactions with minor risk of acute toxicity or treatment failure that typically require standard observation; a +2 weight signifies moderate interactions causing noticeable exposure or physiological fluctuations that neccessitate clinical monitoring or dose adjustment; and a +3 weight is reserved for potent or severe interactions, such as profound exposure changes or life-threatening clinical toxicities, that require immediate intervention or strict avoidance.

**TABLE 1:** The three-pillar scoring system.

Pillar	Scoring criteria	Points
Pharmacokinetic (PK)	Strong increase/decrease in serum concentration (CYP or non-CYP mediated)	+3
	Moderate increase/decrease in serum concentration (CYP or non-CYP mediated)	+2
Pharmacodynamic (PD)	Severe additive myelosuppression, nephrotoxicity or hepatotoxicity	+3
	Major additive QT prolongation	+3
	Moderate additive myelosuppression, nephrotoxicity, hepatotoxicity	+2
	Additive neurotoxicity	+2
	Minor additive toxicities	+1
	Minor QT prolongation	+1
	Additive electrolyte disturbances	+1
	Additive glucose homeostasis disturbances	+1
Pharmacomicrobiomics (PM)	Inhibition of prodrug activation	+3
	Disruption of enterohepatic recirculation	+2

The pharmacokinetic pillar was stratified by the degree of fluctuation in serum concentration. A weight of +3 was assigned to potent interactions (either CYP or non-CYP mediated) resulting in a strong increase or decrease in bioavailability. A moderate increase/decrease in serum concentration was given +2 points. For immunomodulators identified as having an NTI, PK scores were calibrated against the severity of serum concentration changes. For instance, interactions resulting in a ≥200% increase or ≥50% decrease in the Area Under the Curve (AUC) or C_max_, or those involving potent CYP450 inhibitors/inducers were assigned +3 points. Moderate changes were defined as fluctuations between 50% and 200% of baseline exposure requiring clinical monitoring but usually not resulting in acute toxicity or treatment failure.

From a pharmacodynamic point of view, a more extensive list was defined, stratifying additive toxicities. Severe additive myelosuppression, nephrotoxicity and hepatotoxicity and major QTc prolongation or known arrhythmias, were weighted at +3 points due to the extensive burden they might cause. More specifically, major QT prolongation was defined as drug pairs with known risk of Torsades de Pointes (TdP) or those documented to extend the QTc interval beyond 500 m (ICH E14, 2005). Additive neurotoxicity and moderate additive myelosuppression, nephrotoxicity or hepatotoxicity were both assigned +2 points. Minor additive toxicities affecting skeletal and dermatological adverse, minor QTc prolongation, additive electrolyte disturbance and additive glucose homeostasis disturbance were assigned +1 point.

Finally, within the pharmacomicrobiomic domain, gut-mediated interactions were covered. Inhibition of prodrug activation was weighted at +3 points, while the disruption of enterohepatic recirculation was assigned +2 points, reflecting the significant impact on steady-state serum levels. Moreover, a weight of 2 points was assigned to broad-spectrum antibiotics that diminish the gut microbiome responsible for the pre-systemic metabolism of calcineurin inhibitors, thereby increasing bioavailability. Furthermore, the PM score also accounted for the route of administration, antibiotics used exclusively parenterally, thereby bypassing the gut and sparing the microbiome, were assigned a PM score of 0 unless evidence suggested otherwise.

### 2.4 Risk categorization

To objectively determine the composite risk for each drug-drug interaction pair, the obtained scores from the three mechanistic pillars were summed to produce a Total Risk Score (TRS). Based on this result, drug pairs were categorized in one of the following three groups:

- Low Risk (0–1): Interactions with minimal anticipated impact on patient status, therapy may be continued under observation.
- Moderate Risk (Jian et al., 2025; Bhagat et al., 2021): Significant interaction, close monitoring, laboratory surveillance or dose adjustment needed.
- High Risk (≥4 OR +3 from one category): Potentially life-threatening interaction or risk of failure of therapy; alternative antibiotic is recommended.

## 3 Results

### 3.1 Descriptive statistics

A total of 64 interaction mechanisms were identified and profiled across eight commonly used immunomodulatory. The most frequently implicated antibiotic classes are Macrolides (n = 11), Fluoroquinolones (n = 10), Rifamycin (n = 7), Amphenicol (n = 6), Oxazolidinones (n = 5), Penicillins (n = 5) and Urinary Antiseptics (n = 5). According to the Lexicomp® rating system, 73.43% (n = 47) of these initial interactions were classified as Category C, 18.75% (n = 12) as Category D, and 7.81% (n = 5) as Category X. The mechanism of potential interactions was driven by PK pathways (48.43%; n = 31) cases, and PD incompatibilities (42.18%; n = 27). Pharmacomicrobiomic interactions accounted for 9.37% (n = 6) of databases’ entries.

Following a thorough verification and literature referencing, the total number of interaction mechanisms across the 64 pairs increased by 65.5%, reaching a final count of 106 (PK = 38; PD = 44; PM = 24), with the PM pillar showing the most significant expansion (Table 2). This increase results in an average 1.65 mechanistic interactions per drug pair, highlighting the multidimensional nature of these interferences. Examples of such discrepancies are shown in Table 3. Following the application of the risk stratification matrix, the average TRS was 3.51 points per interaction (±1.89 SD). Eight interactions were classified as Low Risk, 23 as Moderate Risk and 33 as High Risk.

**TABLE 2:** Statistical comparison of risk assessment.

Metric	Lexicomp®	Three-pillar model	Change (%)
Total interaction mechanisms	64	106	+65.5%
Average mechanism/pair	1.00	1.65	+65%
High-risk classifications	17	33	+94.1%
PK pillar points	32	38	+18.7%
PD pillar points	31	44	+41.9%
PM pillar points	6	24	+300%

**TABLE 3:** Examples of discrepancies.

Immunomodulator	Antibiotic	Lexicomp® rating	TRS	Overlooked mechanism
Tacrolimus	Erythromycin	C	6	Disrupted EHR, minor QT prolongation
Tacrolimus	Ciprofloxacin	C	6	Disrupted EHR, minor QT prolongation
Tacrolimus	Nafcillin	C	5	Disrupted EHR
Cyclosporine A	Erythromycin	C	6	5-fold increase in serum concentration, severe additive nephrotoxicity
Hydroxychloroquine	Clarithromycin	C	6	Inhibited CYP450
Methotrexate	Rifampin	C	5	Additive hepatotoxicity, 50% decrease in serum concentration
Mycophenolate	Linezolid	C	4	Disrupted EHR
Cyclophosphamide	Rifampin	C	5	Additive hepatotoxicity

### 3.2 Calcineurin inhibitors (CNIs)

Tacrolimus and Cyclosporin A were responsible for 40.6% of the 64 drug pairings and 50 mechanistic interactions in total, exhibiting the most complex risk profiles in the study, with a mean TRS of 4.15. The metabolism of these agents is multidimensional, due to their reliance on both hepatic CYP3A4 isoenzymes and microbiome-mediated pre-systemic processing (Peto et al., 2025). This complexity, in addition to their NTI, explains why 65.38% (n = 17) of these interactions were classified as High Risk.

The highest number of interferences were caused by Macrolides with the notable exception of Azithromycin. These agents constantly reached a TRS of 6. From a PK perspective Clarithromycin, Erythromycin, and Roxithromycin are potent inhibitors of CYP3A4, significantly increasing serum concentration of CNIs (Westphal, 2000). Pharmacodynamically speaking, both drug classes potentially prolong the QT interval and finally, due to their broad-spectrum activity and anaerobic microbiome disruption, they constantly weighed 2 points in the PM pillar (Kunasol et al., 2025; Patel and Rout, 2026).

Potent inducers of the CYP3A4 isoenzyme, such as Rifampin and Nafcillin, were categorized as High-Risk interaction (TRS = 5; PK:3 + PM:2), potentially leading to ineffective therapeutic levels (Chattopadhyay et al., 2018; Fahr, 1993). Ciprofloxacin was also included in this category, due to additive QT prolongation and nephrotoxicity, combined with moderate enzymatic inhibition (Briasoulis et al., 2011; Hajji et al., 2018).

Distinctly, the Tacrolimus + Tigecycline drug pair was weighed at a TSR of 2, making it a Moderate-Risk interaction, solely based on the disruption of enterohepatic circulation, increasing the serum concentration of this NTI agent.

### 3.3 Antimetabolites

The antimetabolite class, including Azathioprine, Mycophenolate Mofetil and Methotrexate, accounted for 39% of the total drug pairs and 38 distinct mechanistic interactions. With a mean TRS of 3.16 (Azathioprine = 2.25; Mycophenolate = 3.14; Methotrexate = 3.42) this group exhibited a generally safer interaction profile than CNIs. Nonetheless, 44% (n = 11) drug pairs were classified as High-Risk.

The pharmacodynamic interactions were dominated by additive myelosuppression. Antibiotics with significant bone marrow toxicity, such as Chloramphenicol, Linezolid, Trimethoprim and Sulfametoxazole constantly weighed 3 points in the PD pillar.

Pharmacokinetic incompatibilities were characterized by an increase in the serum concentrations of the immunosuppressants, especially that of Methothrexate, due to a competitive OAT1/3 and BCRP blockage (Hwang et al., 2024).

Pharmacomicrobiomic interactions were identified in 13 drug pairs, specifically affecting Mycophenolate Mofetil and Methothrexate. Both agents were assigned 2 points in the PM pillar when combined with a broad-spectrum antibiotic. In the case of Mycophenolate, this disruption of the enterohepatic recirculation leads to a decrease in serum concentration (Guo et al., 2019). Conversely, the disturbance of the commensal flora results in unpredictable Methotrexate levels, by eliminating the bacteria responsible for local degradation, thereby increasing the risk of adverse effects and toxicity (Yan et al., 2021).

### 3.4 Other immunomodulatory agents

The final group of miscellaneous immunomodulators, including Cyclophosphamide, Hydroxychloroquine and dapsone, accounted for the remaining 20.3% of the studied pairs. This group was characterised primarily by specific pharmacodynamic interactions rather than pharmacomicrobiomic interferences.

Pairs involving Cyclophosphamide were assigned the highest scores for additive myelosuppression. The combination with protein-synthesis inhibitors like Chloramphenicol and Linezolid reached High-Risk status due to the cumulative bone marrow toxicity (Cyclophosphamide, 2003).

Hydroxychloroquine exhibited the safest overall profile, with most pairings classified as Low-Risk. For this drug, the main clinical concerns identified were additive QT prolongation when combined with macrolides and fluoroquinolones, as well as glucose homeostasis disturbances, warranting cautious monitoring rather than dose adjustment (Infante et al., 2020).

Only one drug pair in the study included dapsone (paired with Rifampin). This interaction received a Moderate-Risk score due to Rifampin induced cytochrome P450 enzymes decreasing the serum concentrations of dapsone below therapeutic levels (Gill et al., 1995).

### 3.5 Comprehensive decision-making matrix

**FIGURE 1:**
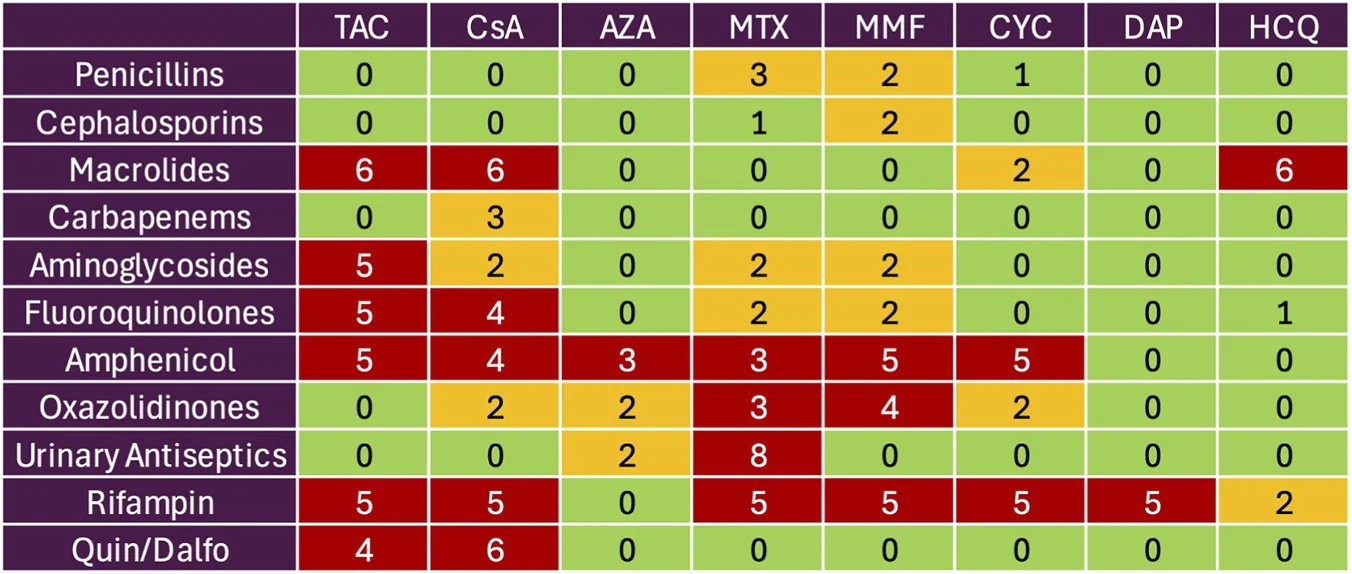
Clinical decision-making matrix for immunomodulator and antibiotic coadministration TAC = tacrolimus, CsA = cyclosporin A, AZA = azatioprine, MTX = methotrexate, MMF = mycophenolate mofetil, CYC = cyclophosphamide, DAP = dapsone, HCQ = hydroxychloroquine * Nafcillin was identified as the only dangerous penicillin in our study, being a potent inducer of CYP3A4.

To facilitate the clinical decision-making process, the data from the 106 analyzed interaction mechanisms were used in the synthesis of a comprehensive risk matrix. This table cross-references the eight immunomodulators against the studied antibiotic classes, using color coding based on the TRS to provide a visual tool. Figure 1 highlights High-Risk interactions in red, Moderate-Risk interactions in yellow and Low-Risk interactions in green.

## 4 Discussion

### 4.1 The importance of the three-pillar approach

The objective of this study was to expand the traditional two-dimensional paradigm of drug-drug interactions and introduce pharmacomicrobiomics as a third, equally important pillar of the topic. While Lexicomp® and other clinical tools described each drug pairing with a single mechanistic interaction, our study found that there is an average of 1.65 interactions per drug pair, representing a substantial expansion of the known interaction landscape.

Relying on two-dimensional tools that fail to recognize multiple interaction pathways for a single drug pair is not only dangerous by creating a false sense of clinical safety, but also implies simple solutions, such as dose adjustment, when the problem would normally require a more complex approach. For instance, the Methotrexate and Sulfonamide pair was originally classified as a Category D interaction due to additive myelosuppression, a pharmacodynamic interaction. However, upon further examination, the same drug pair received five additional points due to significant mechanisms that have been overlooked. This risk of life-threatening cytopenias is further increased by sulfonamides decreasing the renal clearance of Methotrexate, as well as diminishing the gut bacteria responsible for the enterohepatic degradation of this agent (Tan et al., 2022). Ignoring these two crucial pathways underestimates the risk of this combination, which, according to our score, is a High-Risk interaction in contrast to the category assigned by Lexicomp®. Indeed, the literature research confirmed that in some cases, the consequences could be life threatening, certain presentations reporting emergency department visits, severe pancytopenia and acute renal insufficiency caused by this combination (Hamid et al., 2018).

Another striking example is the Tacrolimus-Levofloxacin pairing. Levofloxacin is often considered one of the safest agents within the fluoroquinolone class due to its negligible effects on the CYP450 enzyme family, in contrast to Ciprofloxacin, a potent inhibitor (Zhang et al., 2008). Due to this lack of interference with the hepatic enzymes, Lexicomp® classified this pair as a Category C interaction based only on a pharmacodynamic concern, both agents potentially prolonging the QT interval. This reductive approach overlooked a crucial pharmacomicrobiomic interference. As a broad-spectrum antibiotic, Levofloxacin significantly depletes the commensal gut flora responsible for the enteric degradations of Tacrolimus (Bernard et al., 2016). When this break-down is eliminated, Tacrolimus concentrations fail to decline as expected, leading to elevated systemic levels and an increased risk of nephrotoxicity, QT prolongation and of the adverse effects.

### 4.2 Pharmacomicrobiomics

Importantly, the impact of antibiotic-induced dysbiosis is not consistent across all immunomodulators, as it primarily depends on the drug’s metabolic pathway. Therefore, the clinical consequences of concomitant broad-spectrum antibiotherapy can range from sub-therapeutic levels to toxic surges in serum concentrations. Mycophenolate mofetil undergoes a process called enterohepatic recirculation, which involves the reactivation of its metabolites by intestinal bacterial β-glucoronidases, responsible for roughly 40% of total drug exposure (Saqr et al., 2025). When, during a course of antibiotics, the commensal gut flora is diminished, this reactivation is halted, leading to insufficient serum concentrations and an increased risk of disease flares or graft rejection.

On the contrary, the commensal bacterium *Faecalibacterium prausnitzii* is partially responsible for the breakdown of Tacrolimus into a metabolite 15 times less potent than the parent drug (Guo et al., 2019). Patients receiving broad-spectrum antibiotics likely experience a decline in this specific microbial population, resulting in an impaired breakdown that drives serum concentrations toward toxic levels, significantly increasing the risk of adverse effects such as acute kidney injury.

### 4.3 Safest alternatives

Departing from the High-Risk interactions, certain antibiotics, specifically Azithromycin, Penicillins and Cephalosporins constantly occupied the safe end of the spectrum, representing the ‘green zone’ of the risk matrix.

In contrast to other macrolides, Azithromycin does not inhibit the CYP3A4 isoenzyme, ensuring stable serum concentrations during antibiotherapy. From a pharmacokinetic perspective, it poses minimal threat to most immunomodulator treatments, with its only High-Risk classification occurring when paired with Hydroxychloroquine due to synergistic risk of significant QT prolongation.

Similarly, penicillins and cephalosporins generally received Low-to Moderate-Risk classifications. In most cases, the identified interactions were predominantly driven by the disruption of enterohepatic recirculation or additive hypokalemic effects, the latter of which warrants cautious monitoring of electrolyte levels rather than dosage adjustment or drug modification (Peto et al., 2025; Seo and Kim, 2022).

Notably, Nafcillin was identified as the only dangerous penicillin in our study, being a potent inducer of CYP3A4, potentially leading to sub-therapeutic exposure and treatment failure (Lang et al., 2003).

### 4.4 Limitations of the study and the next phase

Despite the rigorous mechanistic framework provided by the three-pillar model, several limitations must be acknowledged. Primarily, the theoretical nature of this study means that the risk scores are based on existing literature and established drug-drug interaction checkers rather than direct clinical observation. While the matrix serves as a comprehensive decision-making tool, complex interactions in multimorbid patients may yield outcomes that differ from *in silico* predictions.

Another primary limitation of this study is the reliance on the Lexicomp database for the initial identification of DDIs. While it is an internationally recognized DDI checker, it is not exhaustive. Consequently, our analysis is constrained by this database, any potential interactions not currently indexed in Lexicomp were inherently excluded from our study.

Secondly, the pharmacomicrobiomic pillar of the risk score is inherently contingent upon the high degree of inter-individual variability in gut microbiome composition. Just as genetic polymorphism influences the activity of the cytochrome P450 enzyme family, factors such as diet, geography, and prior antibiotic exposure dictate the commensal gut flora of a patient, potentially affecting the intensity of the upper mentioned interactions.

The next phase of this research involves the clinical validation of the developed risk score. We intend to conduct a retrospective cohort study within a rheumatology clinic to correlate our predicted TRS with actual adverse effects, clinical outcomes and fluctuations in therapeutic drug levels. Future research could include machine learning algorithms, such as Random Forest, or XGBoost, on larger databases, to evaluate the predictive accuracy of this proposed scoring system against real clinical outcomes.

## 5 Conclusion

Conventional drug-drug interaction checkers are predominantly two-dimensional, overlooking nearly 40% of mechanistic pathways. This study demonstrates that a three-pillar model (PK, PD, PM) is essential for the comprehensive understanding of immunomodulator-antibiotic safety. By identifying a 65.5% increase in mechanistic interactions, we have highlighted a significant clinical knowledge gap that negatively affects both therapeutic efficacy and patient safety. As the role of the gut microbiome gains increasing recognition in medicine, pharmacomicrobiomics must be integrated into therapeutic decision-making. This advancement is necessary to ensure the safe and effective use of immunomodulators and the optimized treatment of bacterial infections in this highly vulnerable patient group.

## Data Availability

The original contributions presented in the study are included in the article/Supplementary Material, further inquiries can be directed to the corresponding author.
